# Combined atezolizumab and nab-paclitaxel in the treatment of triple negative breast cancer: a meta-analysis on their efficacy and safety

**DOI:** 10.1186/s12885-022-10225-y

**Published:** 2022-11-05

**Authors:** K. Sharmni Vishnu, Thin Thin Win, Saint Nway Aye, Arun Kumar Basavaraj

**Affiliations:** 1 BMed Science, School of Medicine, International Medical University, 126, Jalan Jalil Perkasa 19, Bukit Jalil, 57000 Kuala Lumpur, Malaysia; 2 Adelaide Medical School, The University of Adelaide, North Terrace, Adelaide, 5005 Australia; 3 Pathology Department, School of Medicine, International Medical University, 126, Jalan Jalil Perkasa 19, Bukit Jalil, 57000 Kuala Lumpur, Malaysia

**Keywords:** Triple negative breast cancer, Atezolizumab, PD-L1 inhibitor, systematic review, and meta-analysis

## Abstract

**Background:**

Triple negative breast cancer (TNBC) is clinically aggressive breast cancer with a poor prognosis. Approximately 20% of TNBC has been found to express programmed death ligand 1 (PD-L1), making it a potential therapeutic target. As a PD-L1 inhibitor, atezolizumab is a recently approved immunotherapeutic drug for TNBC, this meta-analysis (MA) was aimed to review the randomized controlled trial studies (RCTs) of combined atezolizumab and nab-paclitaxel in the treatment of TNBC and synthesize the evidence-based results on its effectiveness and safety.

**Method:**

We searched PubMed, Embase, EBSCOhost and ClinicalTrials.gov for the eligible RCTs which compared the efficacy and safety of combined atezolizumab and nab-paclitaxel with nab-paclitaxel alone. The outcomes analyzed included overall survival (OS), progression-free survival (PFS), objective response rate (ORR) and treatment-related adverse effects (AEs).

**Results:**

A total of six RCTs were included in this MA. For efficacy, although OS was not significantly prolonged with combined atezolizumab and nab-paclitaxel (HR 0.90, 95% CI [0.79, 1.01], *p*=0.08), this combination therapy significantly improved PFS (HR 0.72, 95% CI [0.59, 0.87], *p*=0.0006) and ORR (RR 1.25, 95% CI [0.79, 1.01] p<0.00001). For safety, any AEs, haematological, gastrointestinal, and liver AEs showed no statistically significant differences between the atezolizumab and nab-paclitaxel combination group and nab-paclitaxel alone group. However, serious AEs, high grade, dermatological, pulmonary, endocrine, and neurological AEs were significantly lower with nab-paclitaxel alone compared to atezolizumab and nab-paclitaxel combined (*p*-value range from <0.00001 to 0,02).

**Conclusion:**

Atezolizumab combined with nab-paclitaxel was associated with improved outcomes in the treatment of TNBC; however, this combination resulted in more toxicity compared to nab-paclitaxel alone. While nab-paclitaxel alone produced chemotherapy-related AEs, the combination of atezolizumab with nab-paclitaxel produced AEs, especially immune-related AEs such as haematological, pulmonary, endocrine, and neurological AEs.

**Trial registration:**

This research work of systematic review has been registered on PROSPERO (Registration number: CRD42022297952).

**Supplementary Information:**

The online version contains supplementary material available at 10.1186/s12885-022-10225-y.

## Introduction

Triple negative breast cancer (TNBC) is a molecularly diverse breast cancer subtype which shows hormone receptor immunohistochemistry (IHC) stains of less than 1% for oestrogen and progesterone; and the absence of human epidermal growth factor receptor-2 (HER2) protein expression or *HER2* gene amplification or both [1, 2]. It is associated with earlier age of onset, aggressive clinical course and poor prognosis with worse survival [3].

TNBC is usually treated with a combination of surgery, radiotherapy and chemotherapy. The successes of atezolizumab which is a monoclonal antibody-based immune checkpoint inhibitor (ICI) against programmed cell death- ligand 1 (PD-L1) have been achieved in various cancers such as non-small cell lung carcinoma, renal cell carcinoma, urothelial carcinoma [4]. In March 2019, the Food and Drug Administration (FDA) approved atezolizumab for the treatment of people with TNBC. Efficacy of atezolizumab in combination with nab-paclitaxel has been further approved by IMpassion130 Investigators [5].

As atezolizumab is a recently approved immunotherapeutic drug for TNBC, systematic review (SR) and meta-analysis (MA) on its effectiveness and safety in combination with nab-paclitaxel, have not been studied properly in published primary studies. The results of some primary randomized control trial studies (RCTs) and clinical trials showed uncertainties and conflicting results which makes challenging for the clinician to integrate the data into clinical practice. Therefore, this study systematically reviewed the RCTs of combined atezolizumab and nab-paclitaxel in the treatment of TNBC and synthesize the evidence-based results on its effectiveness and adverse effects of it. This MA was also able to report the efficacy of the combined therapy group in association with PD-L1 immunohistochemistry positivity of TNBC.

## Materials and methods

The SR and MA were done according to the updated guideline of the Preferred Reporting Items for Systematic Review and Meta-Analyses (PRISMA) statement [6].

### Identification of eligible studies

The systematic literature search was carried out in health-related electronic databases such as PubMed, Embase and EBSCOhost. Clinical trial studies were also searched at ClinicalTrials.gov. The search terms used were “immune checkpoint inhibitors”, “PD-L1 inhibitor”, “atezolizumab”, and “triple negative breast cancer”. The search was limited to original articles published in the English language up to October 2021. To find out additional studies, reference lists of the original articles were also screened.

### Inclusion and exclusion criteria

Studies selection was based on criteria of PICOS format: (1) Participants: All female patients above 18-year-old who have been diagnosed with TNBC by histopathological and immunohistochemical methods; (2) Intervention: Intravenous Atezolizumab and nab-paclitaxel; (3) Comparisons: placebo and nab-paclitaxel or nab-paclitaxel alone; (4) Outcomes: primary outcomes were overall survival (OS) which is defined as the time from randomization to death from any cause and treatment-related adverse effects (AEs). The secondary outcomes included progression free survival (PFS) which is defined as the time from randomization to cancer progression or death from any cause and objective response rate (ORR). Studies included parallel RCTs reporting on the efficacy and or safety of combined atezolizumab and nab-paclitaxel in the treatment of TNBC. Review articles, case reports, editorials, and studies that used combinations other than nab-paclitaxel were excluded for this MA.

### Literature search and study selection

Two researchers (TTW, SVK) conducted an independent literature search using healthcare electronic databases (PubMed, Embase and EBSCOhost) and ClinicalKey to search for related clinical trials. The articles obtained from the literature search were assessed by two researchers (TTW, SVK) independently. Any disagreements between both researchers were initially discussed between two researchers. If an agreement was not reached, two researchers discussed with a third researcher (SNA) to mediate and finalize. The articles were screened according to the PRISMA flowchart. As for the first stage, related articles were identified and grouped to create a total number of records. These records were screened leading to the removal of review articles and non-relevant articles. The articles that did not fit the inclusion criteria based on abstract and title alone were excluded. Finally, full-text articles were examined to obtain the included studies required for MA.

### Data extraction

Two researchers (TTW, SVK) independently extracted the relevant data from the included studies using piloted data extraction sheet. Discrepancies were discussed thoroughly and finalized with a third researcher (SNA). The data that were extracted included study title, first author and year of publication, trial phase, and number of patients receiving combined atezolizumab and nab-paclitaxel, number of patients receiving nab-paclitaxel alone or combined with placebo, mean age of participants, PD-L1 positivity, a dose of atezolizumab and follow up duration. Outcome data included were OS, PFS, ORR, any AEs, serious AEs, high-grade AE, haematological AEs, gastrointestinal AEs, dermatological AEs, pulmonary AEs, liver AEs, endocrine AEs, and neurological AEs.

### Quality assessment of the included studies

The Cochrane evaluation handbook of randomized controlled trials bias tool was used to evaluate the bias and quality of the included studies. It included the following assessment scopes: random sequence generation, allocation concealment, the blindness of participants and personnel, the blindness of outcome assessment, incomplete outcome data, selective reporting, and other biases. We rated each domain of the tool as having a 'low', 'high', or 'unclear' risk of bias at the study level and for each outcome if possible [7].

### Statistical analysis

Among the outcomes, analysis of OS and PFS was estimated as the hazard ratio (HR) and analysis of other outcomes was estimated as the risk ratios (RR) for the treatment success of combined atezolizumab and nab-paclitaxel vs nab-paclitaxel alone. From the statistical analysis, heterogeneities were assessed using the chi-squared test and the I^2^ statistic. Pearson's chi-squared test was used to decide a statistically significant difference between the expected frequencies and the observed frequencies. To avoid the heterogeneity, if the I^2^ statistic is more than 50%, a random effects model was used; and if the I^2^ statistic is less than 50%, a fixed effect model was used. We investigated the robustness of the review by performing sensitivity analyses when appropriate, such as performing fixed-effect models for selected outcomes and including only low risk of bias outcomes, according to the summary assessment of the risk of bias. A two-tailed P value of less than 0.05 was considered statistically significant. A 95% CI was used to provide a range of values for the ORs obtained. MA has performed with Review Manager (RevMan 5.4) software.

## Results

### Literature search results

**Fig. 1:**
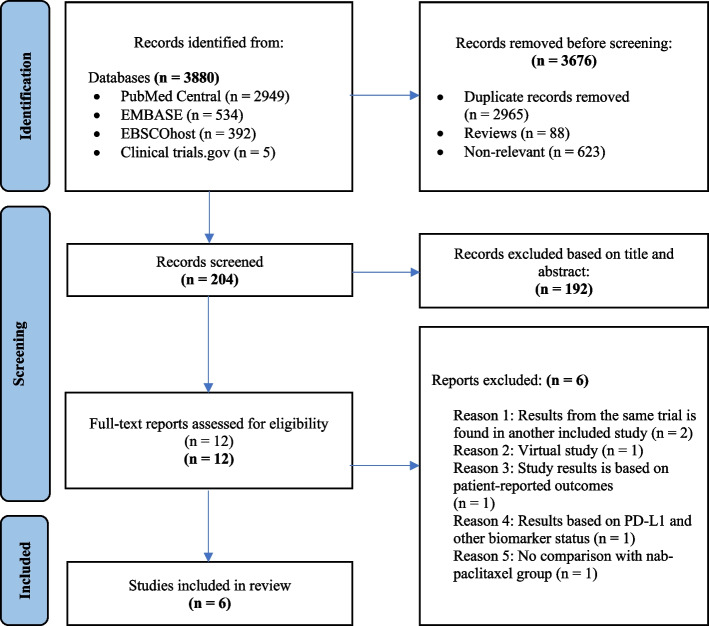
PRISMA flowchart summarizing the process to identify the eligible studies (Adapted from PRISMA 2020 flow diagram for new systematic reviews which included searches of databases and registers only [6])

A total of 3880 potential studies were identified using the preliminary search strategy; 2949 were from PubMed, 534 were from EMBASE, 392 were from EBSCOhost and 5 were clinical trials from ClinicalKey. A total of 2965 studies were compiled after removing the duplicates. Based on the titles, 88 review articles and 623 irrelevant studies were removed. After screening abstracts of the remaining 204 studies, 192 studies were excluded as they were not specifically related to our specific search criteria. Finally, 12 studies were accessed for eligibility by reading the full text. Among them, 6 studies were excluded with reasons based on inclusion and exclusion criteria. Therefore, the remaining 6 articles were included for SR and MA. A three-phase flow chart of the study selection process based on the updated PRISMA statement 2020 is illustrated in Fig. 1. A summary of the reasons for six excluded studies [8–13] is shown in Table S1.

### Characteristics of the included studies

The characteristics of the six included studies [5, 14–18] are shown in Table 1. In all six included studies, the dosage of atezolizumab was 840 mg given intravenously on days 1 and 15, and chemotherapy was administered every week on days 1, 8 and 15: with a follow-up duration of 2 years. Five studies were phase 3 RCTs and 1 was phase 2 RCTs. These 6 studies include a total of 1078 study participants in the intervention arm and 886 participants in the control arm. Out of these participants, 837 had breast cancer cells which were PD-L1 positive. The first two studies by Schmid referred to the same study with a follow-up publication. The first article had data to assess the efficacy of the treatment whereas the updated article published in 2020 had more comprehensive data on the AEs which is used to assess the safety of this drug combination therapy [5, 14].

**Table 1:** Characteristics of included studies

Study, Year [Ref]	Trial	Phase of study	Atezolizumab + Chemo (Intervention) (n)	Placebo + Chemo (Control) (n)	PD-L1 positive cases (n)	Mean age (range)	Dose of Atezolizumab	Follow-up duration (months)
Schmid 2018 [14]	NCT02425891	II	451	451	368	55 (20-82)	840 mg, IV on days 1 and 15	33
Schmid 2020 ([5]	NCT02425891	III	451	451	369	55 (46-64)	840 mg, IV on days 1 and 15	42
Iwata 2019 [15]	NCT02425891	III	34	31	25	57 (31-82)	840 mg, IV on days 1 and 15	24
Mittendorf 2020 [16]	NCT03197935	III	165	168	154	51 (22-76)	840 mg, IV on days 1 and 15	24
Miles 2021 [17]	NCT03125902	III	431	220	292	54 (22-85)	840 mg, IV on days 1 and 15	24
Brufsky 2021 [18]	NCT02322814	II	31	47	22	51 (20-75)	840 mg, IV on days 1 and 15	24

### Assessment of risk of bias and publication bias

**Fig. 2:**
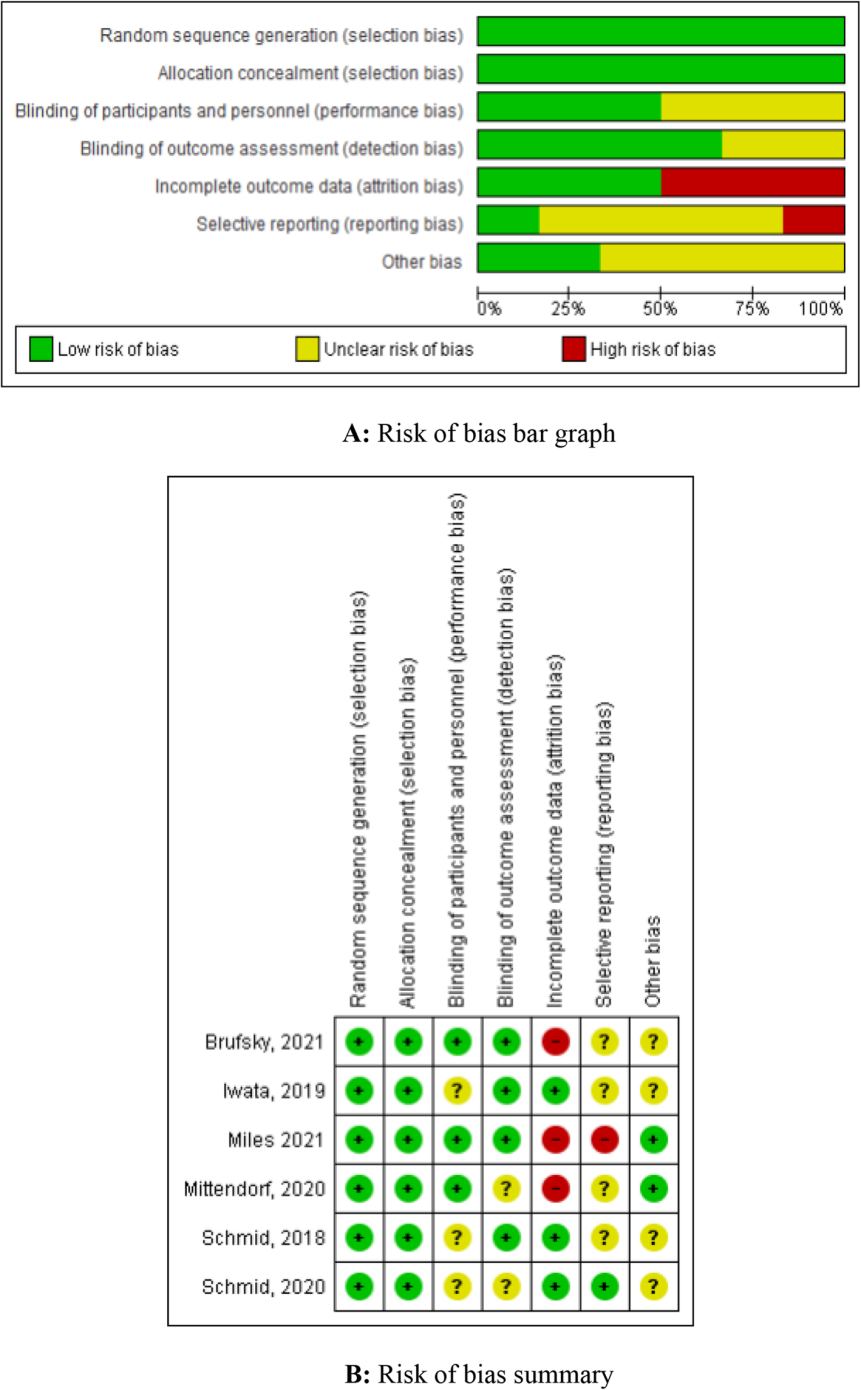
**A** Risk of bias bar graph. **B** Risk of bias summary

The results of the assessment of the bias are shown in the risk of bias graph (Fig. 2A) and the risk of bias summary (Fig. 2B). The overall risk of bias was evaluated as low risk and the quality of the studies was acceptable. Publication bias was assessed in six included studies. Begg’s and Egger’s test Funnel plot for publication bias is shown in Figure S1.

### Efficacy of atezolizumab for TNBC

**Fig. 3:**
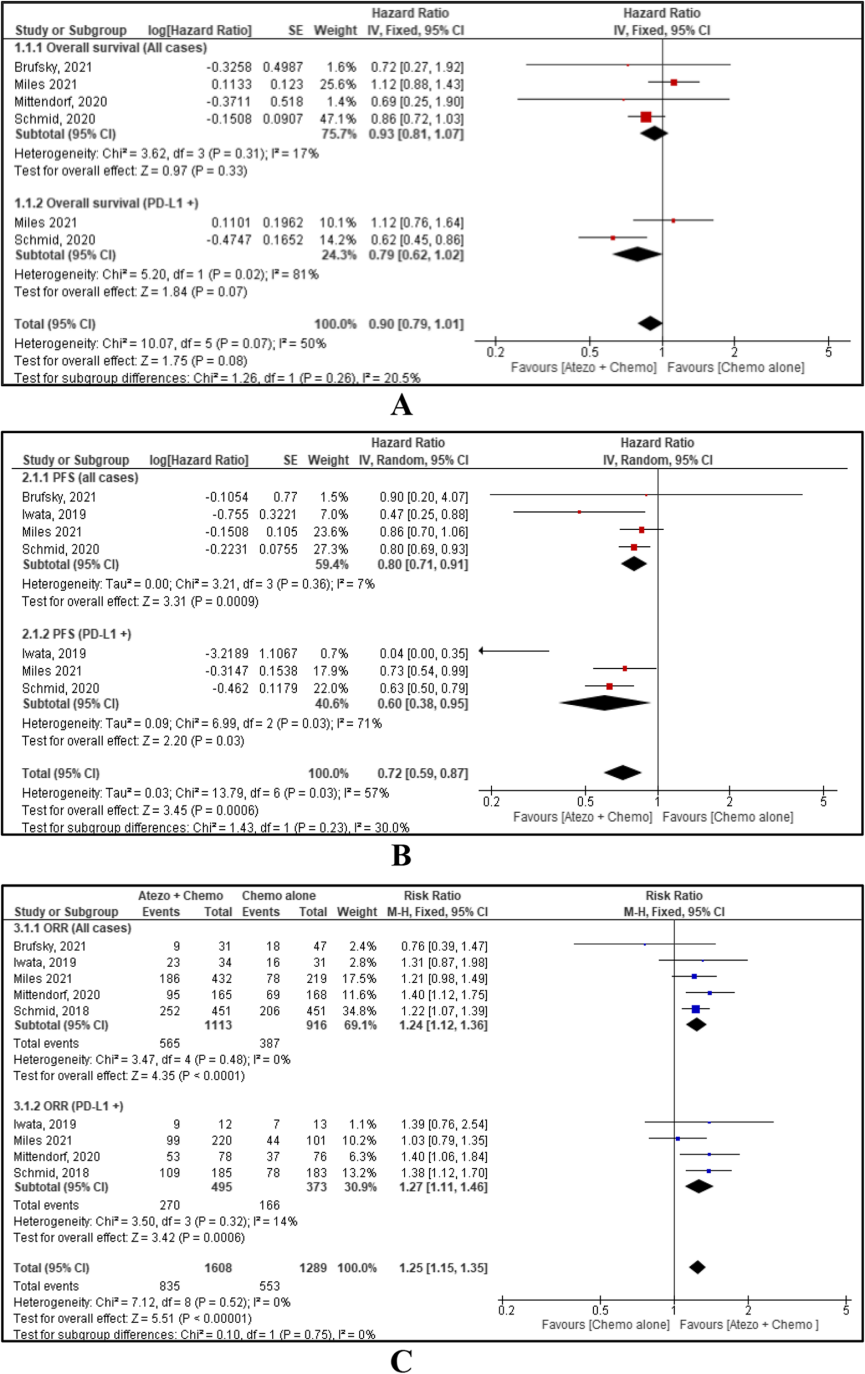
Forest plot and meta-analysis results for OS (**A**); PFS (**B**); ORR (**C**)

As included studies reported OS, PFS and ORR for all TNBC cases and PD-L1 positive cases, this MA performed a sub-group analysis on it. OS was reported by four studies [5, 16–18]. The HR for OS in the all-cases group, and in the PD-L1 positive subgroup were 0.93 and 0.79 respectively with HR 0.90, 95% CI [0.79, 1.01], *p*=0.08. There is no evidence of a difference in OS between combined atezolizumab with chemotherapy and chemotherapy alone (Fig. 3A). PFS was reported by four studies [5, 15, 17, 18]. Combined atezolizumab with chemotherapy for both all cases and PD-L1 positive cases showed favourable PFS with HR 0.72, 95% CI [0.59, 0.87], *p*=0.0006 (Fig. 3B). ORR was reported by five studies [14–18], and favourable ORR was seen with combined atezolizumab and chemotherapy. Analysis on all-cases group and PD-L1 positive subgroup showed RR 1.24, 95% CI [1.12, 1.36], p<0.0001 and RR 1.27, 95% CI [1.11, 1.46], *p*=0.0006 respectively, and overall RR 1.25, 95% CI [1.15, 1.35], p<0.00001 (Fig. 3C).

### Safety of atezolizumab for TNBC

**Fig. 4:**
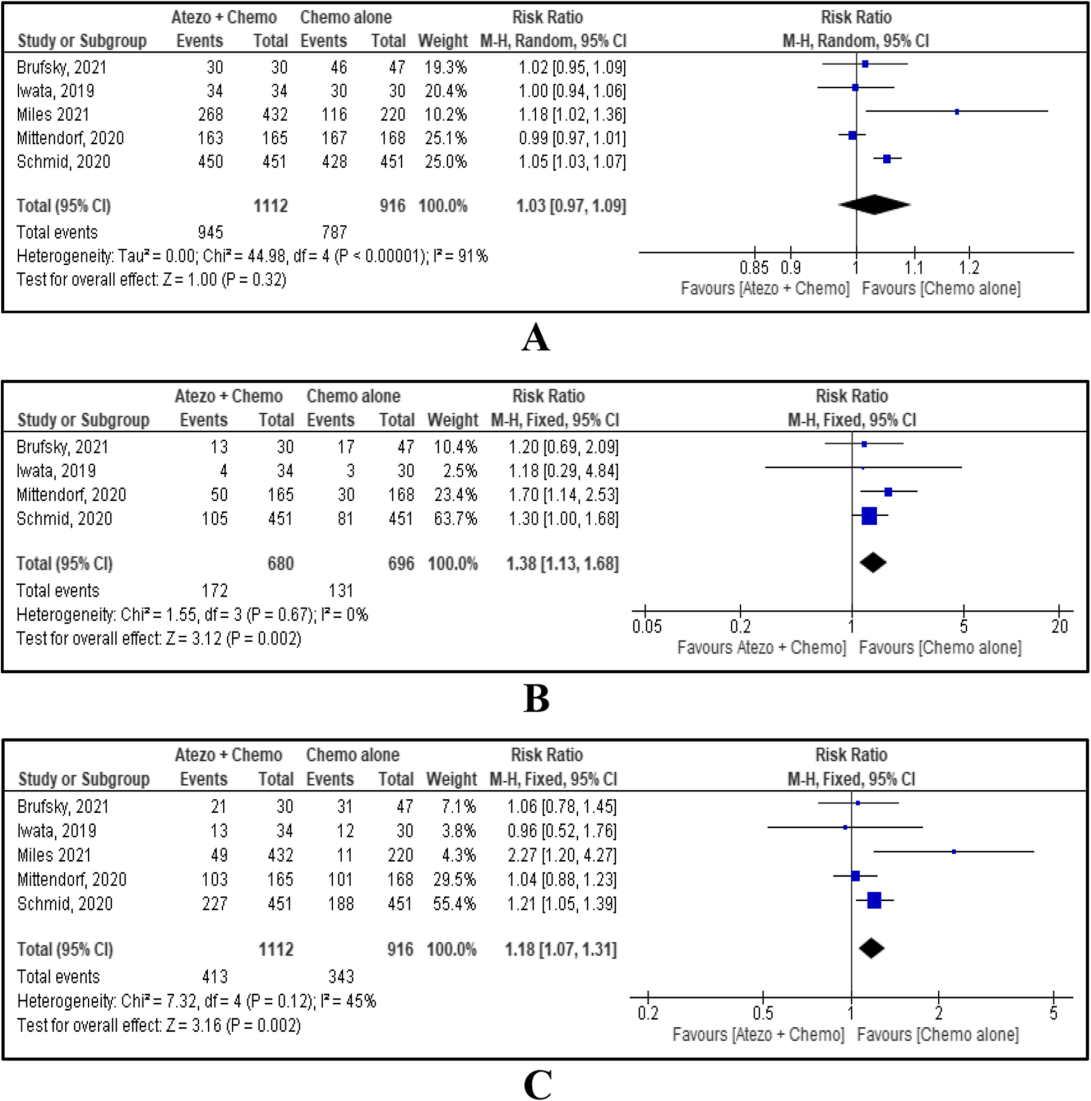
Forest plot and meta-analysis results for any AEs (**A**); serious AEs (**B**); high-grade AEs (**C**).

Incidences of various AEs comparing combined atezolizumab and chemotherapy Vs chemotherapy alone are shown in Table 2. In this MA, any treatment-related AEs and high-grade AEs were reported by five included studies [5, 15–18], and serious AEs were reported by four studies [5, 15, 16, 18]. Incidence of any AEs between the two groups showed no evidence of a difference with RR 1.03, 95% CI [0.97, 1.09], *p*=0.32 (Fig. 4A). Incidence of serious AEs and high-grade AEs were significantly increased with atezolizumab and chemotherapy combination therapy with RR 1.38, 95% CI [1.13, 1.68], *p*=0.002 and RR 1.18, 95% CI [1.07, 1.31], *p*=0.002 respectively. (Fig. 4B, C)

**Table 2:** Incidence of various AEs comparing combined atezolizumab + chemotherapy and chemotherapy alone

Adverse Effects (AEs)	No. of studies	RR (95% CI)	p-value	I^**2**^ (%)	Statistical method
Any AEs	5	1.03 (0.97, 1.09)	0.32	91	Random effect
Serious AEs	4	1.38 (1.13, 1.68)	0.002	0	Fixed effect
High grade AEs	5	1.18 (1.07, 1.31)	0.002	45	Fixed effect
Haematological AEs	5	1.10 (0.98, 1.23)	0.10	50	Random effect
Gastrointestinal AEs	5	1.06 (0.89, 1.27)	0.49	72	Random effect
Dermatological AEs	5	1.16 (1.02, 1.31)	0.02	6	Fixed effect
Pulmonary AEs	4	1.36 (1.09, 1.71)	0.007	44	Fixed effect
Liver AEs	5	1.13 (0.94, 1.35)	0.18	30	Fixed effect
Endocrine AEs	4	3.77 (2.78, 5.11)	<0.00001	0	Fixed effect
Neurological AEs	5	1.13 (1.00, 1.26)	0.04	0	Fixed effect

**Fig. 5:**
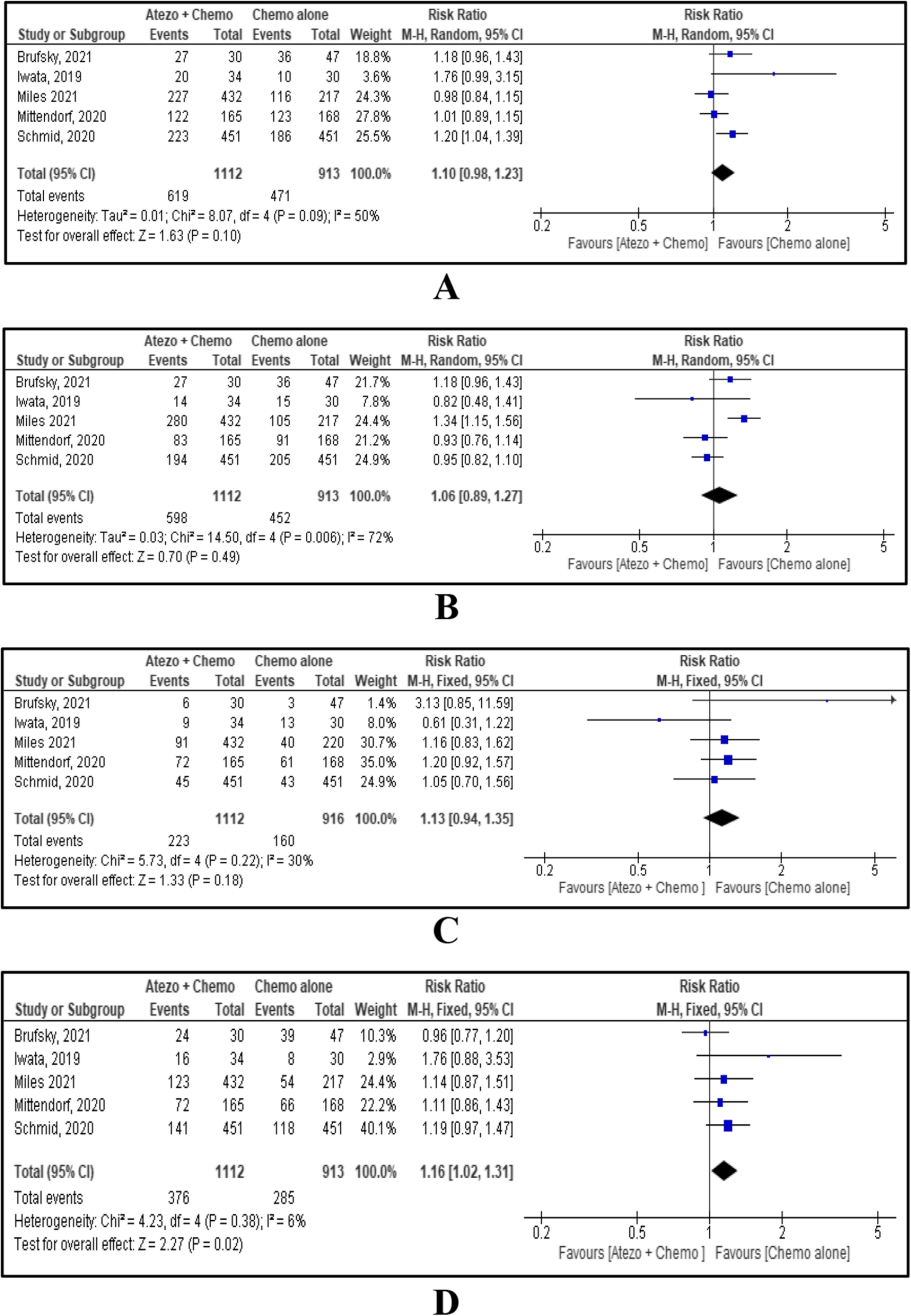
Forest plot and meta-analysis results for haematological AEs (**A**); gastrointestinal AEs (**B**); liver AEs (**C**), dermatological AEs (**D**)

Haematological, gastrointestinal, liver, and dermatological AEs were reported by five studies [5, 15–18]. It did not show any statistically significant difference in the safety of atezolizumab combination therapy compared to chemotherapy alone with RR 1.10, 95% CI [0.98, 1.23], *p*=0.10, RR 1.06, 95% CI [0.89, 1.27], *p*=0.49 and RR 1.13 95% CI [0.94, 1.35], *p*=0.18 for Haematological, gastrointestinal, liver AEs respectively (Fig. 5A, B, C). However, dermatological AEs were statistically increased with combined atezolizumab and chemotherapy with RR 1.16, 95% CI [1.02, 1.31], *p*=0.02). (Fig. 5D)

**Fig. 6:**
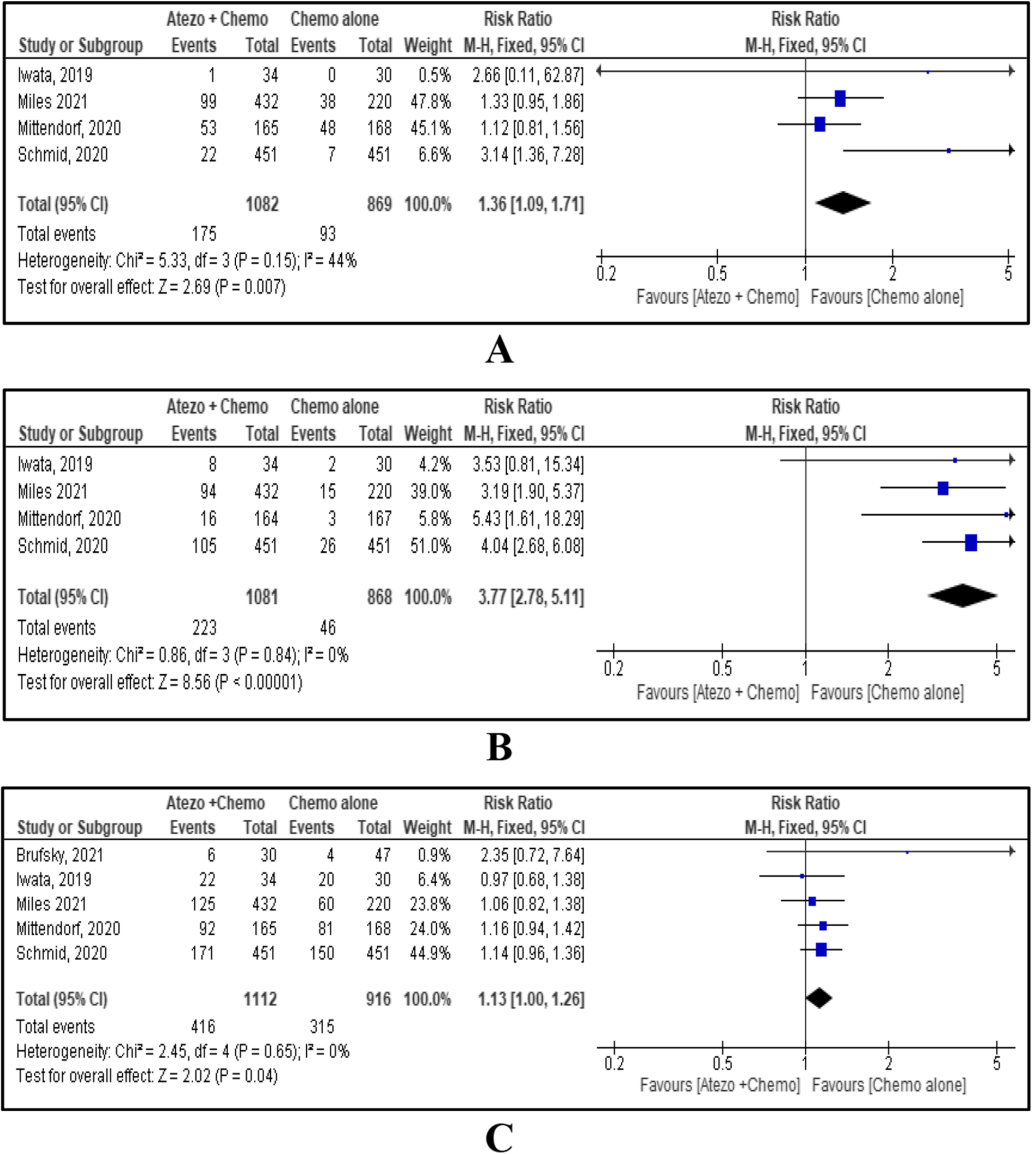
Forest plot and meta-analysis results for pulmonary AEs (**A**), endocrine AEs (**B**); neurological AEs (**C**)

Pulmonary and endocrine AEs were reported by four studies [5, 15–17]. Pulmonary AEs were not significantly different between the two treatment groups with RR 1.36, 95% CI [1.09, 1.71], *p*=0.007 (Fig. 6A). However, endocrine AEs were statistically significant increased with combined atezolizumab and chemotherapy with RR 3.77, 95% CI [2.78, 5.11], p<0.00001 (Fig. 6B). Neurological AEs were reported by five studies [5, 15–18] and it was significantly increased with combined atezolizumab and chemotherapy with RR 1.13, 95% CI [1.00, 1.26], *p*=0.04 (Fig. 6C).

## Discussion

In recent years, the role of immunotherapy in cancer treatment has expanded and now it is the first choice of treatment intervention in many cancers [19]. ICIs have demonstrated favourable outcomes in a variety of refractory solid malignancies such as advanced non-small cell lung cancer, metastatic melanoma, and metastatic bladder cancer. Unlike these tumour types, most breast cancers are not inherently immunogenic, however, TNBC is the most immunogenic subtype [20]. TNBC has been found to have higher rates of cell surface PD-L1 expression compared to other breast cancer subtypes and higher PD-L1 expression suggests a greater potential benefit from the use of PD-1/PD-L1 targeted immunotherapy [20, 21].

Atezolizumab which is anti-PD-L1 antibody was approved to be used in combination with nab-paclitaxel, mainly for PD-L1 expressed locally advanced or metastatic TNBC as determined by an FDA-approved test. The FDA also approved the VENTANA PD-L1 (SP142) Assay as a companion diagnostic device for selecting TNBC patients for atezolizumab [22].

In this MA, all included studies used VENTANA PD-L1 (SP142) immunohistochemical assay for the determination of PD-L1. Determination of PD-L1 status is indication-specific, and evaluation is based on either the proportion of tumor area occupied by PD-L1 expressing tumor-infiltrating immune cells (% IC) of any intensity or the percentage of PD-L1 expressing tumor cells (% TC) of any intensity. According to diagnostic indications for the VENTANA PD-L1 (SP142) Assay by ROCHE VENTANA, cutoff score for TNBC is ≥ 1% IC; whereas ≥ 50% TC or ≥ 10% IC for non-small cell lung cancer and ≥ 5% IC for urothelial carcinoma [23, 24]. Routine testing for PD-L1 immune-cell expression in unresectable, locally advanced or metastatic TNBC with the approved companion diagnostic (VENTANA PD-L1 SP142 assay) should be used to identify patients who might benefit from treatment with atezolizumab plus nab-paclitaxel [5].

### Efficacy combined atezolizumab and nab-paclitaxel for TNBC

Based on our best literature search, MA on the efficacy of combined atezolizumab and nab-paclitaxel versus nab-paclitaxel alone in the treatment of TNBC has not been published. All the MA on efficacy were done on all ICIs. In our MA, efficacy is significantly improved when atezolizumab is added to chemotherapy for the treatment of TNBC based on a pooled analysis of ORR, although there is no significant difference OS and PFS between the two groups. However, PFS in PD-L1 positive group showed slightly favourable PFS compared with all cases and ORR in PD-L1 positive group showed less favourable ORR compared with all cases.

The results of this MA are also concordant with other published MAs on combined ICIs. A MA on the efficacy of PD-1/PD-L1 inhibitors in TNBC reported significant anti-tumour effect with combined PD-1/PD-L1 inhibitors and chemotherapy proven by OS and ORR [25]. Efficacy of combined neoadjuvant chemotherapy and ICIs based on pathological complete response (pCR) and tumour characteristics were reported by a MA on early stage TNBC. It reported improved pCR and PFS in the group with combined ICIs and neoadjuvant chemotherapy [26]. In that study, response to ICIs depending on PD-L1 status was also assessed, and it was found that both PD-L1 positive and PD-L1 negative patients had no statistically significant difference in their treatment outcomes. One possible explanation for this could be that the study participants in this study were patients with early-stage TNBC. The immune cell dynamics in the tumour micro-environment in the early stages and late stages of TNBC are substantially different, and a subgroup analysis of the PD-L1 status is still important and valid [27]. Latest MA on atezolizumab and pembrolizumab in TNBC reported that ORR of atezolizumab/pembrolizumab plus chemotherapy was higher in the intention to treat arms than the placebo groups in TNBC. This study also collate evidence of atezolizumab/pembrolizumab as viable therapeutics among patients with TNBC with PD-L1 subgroups deriving higher benefits [28].

A clinical trial, IMpassion 130 which is a phase 3 trial reported favourable OS with combined atezolizumab and nab-paclitaxel in PD-L1 positive TNBC. However, this positive result could not be formally tested due to the prespecified statistical testing hierarchy [5]. It also prolonged PFS in both intention-to-treat population and PD-L1–positive subgroup. In IMpassion 130, the median PFS in PD-L1 positive patients was 7.5 months in the atezolizumab plus nab-paclitaxel group versus 5.0 months in the placebo plus nab-paclitaxel group [14]. However, these results were contradicted by the results of IMpassion 131 which reported that combining atezolizumab with paclitaxel did not improve PFS or OS versus paclitaxel alone in PD-L1 positive group [17]. Therefore, PD-L1 expression has not consistently correlated with response to immunotherapy in clinical trials, and it cannot yet be considered a meaningful predictive biomarker. Other biomarkers such as tumour mutational burden and tumour infiltrating lymphocytes are now being explored as PD-L1 independent predictive biomarkers for immune responsiveness of the tumour in immunotherapy of TNBC [20].

A MA on the efficacy of atezolizumab for non-small cell lung cancer (NSCLC) found that both high PD-L1 expression and negative PD-L1 expression subgroups had an improved efficacy with atezolizumab. Interestingly, in patients with low expression of PD-L1, OS showed no significant difference between atezolizumab treatment group and the placebo group though the results favoured atezolizumab in terms of PFS [29]. Therefore, atezolizumab combination therapy has its benefits regardless of the PD-L1 status. Although atezolizumab is a PD-L1 inhibitor which binds to PD-L1 on the tumour cells, it is evident that the treatment of cancers with atezolizumab is efficacious even when PD-L1 is absent. Currently, the US FDA has approved atezolizumab to be used with chemotherapy for PD-L1 positive patients in both NSCLC and TNBC [22, 30]. These results could pave the path toward the drug approval of atezolizumab regardless of PD-L1 status in cancers including NSCLC and TNBC.

Studies on the use of atezolizumab as a combination therapy in urothelial carcinoma and hepatocellular carcinoma also showed that the efficacy of atezolizumab combination therapy is greater than chemotherapy alone or atezolizumab monotherapy [31, 32]. This effect is due to the combination of atezolizumab with chemotherapy improving the immunological conditions in the tumour micro-environment which enhance the anti-tumour immune response [33].

### Safety of atezolizumab for TNBC

ICIs can cause immune-related AEs as the mechanism of ICI action relies on the inhibition of the physiological brake of immune activation and they often have off-target effects resulting in immune-mediated inflammation of diverse organs or tissues [34].

In this MA, we analyzed common treatment-related AEs caused by atezolizumab plus nab-paclitaxel versus nab-paclitaxel alone. The AEs analyzed in this MA were any treatment-related AEs, serious AEs, high-grade AEs, haematological, gastrointestinal, dermatological, pulmonary, liver, endocrine and neurological AEs. There is a significant greater incidence of serious AEs, high-grade AEs, dermatological, endocrine, and neurological AEs in the group treated with combined atezolizumab and nab-paclitaxel group compared to chemotherapy alone. However, there are no significant differences in any treatment-related AEs, haematological, gastrointestinal, pulmonary, and liver AEs between the two groups of treatment.

The results on the safety of our MA are concordant with other MA. A MA on the safety of PD-1/PD-L1 inhibitors also showed a higher frequency of AEs in the atezolizumab combination therapy group compared to the placebo control group. Specific immune-mediated AEs above grade 3 highlighted in this study were neurological AEs like peripheral neuropathy and haematological AEs like anaemia and neutropenia [25]. An RCT study on atezolizumab plus nab-paclitaxel in non-small cell lung cancer showed a higher incidence of high-grade AEs in this combination group compared with chemotherapy alone [35]. Other studies have also shown that most AEs of atezolizumab are immune-related, mainly dermatological, gastrointestinal, pulmonary, endocrine and neurological AEs [36]. When comparing the safety of atezolizumab with chemotherapy, cytotoxic AEs are more serious with chemotherapy, producing chemotherapeutics-related deaths [37, 38].

There were some limitations during the conduct of this MA such as a small sample size in some of the primary studies and ongoing trials without reported results. There was also a discrepancy among clinical trials. Although IMpassion 130 used nab-paclitaxel, IMpassion 131 used paclitaxel. In the MA of the efficacy of atezolizumab regarding PD-L1 status, we could manage to perform a sub-group analysis of overall cases and PD-L1 sub-group. Likewise for safety, subgroup analysis of the AEs in the PD-L1 positive population could not be assessed since the primary studies did not provide sufficient data. After the conduct of this MA, the manufacturer of atezolizumab (Roche) has withdrawn the indication for atezolizumab in combination with nab-paclitaxel in the treatment of TNBC following consultation with the FDA in the United States to reassess the status of systemic therapy agents granted accelerated approval [39].

In conclusion, there is evidence of increased efficacy in combining atezolizumab with chemotherapy for the treatment of TNBC. Furthermore, atezolizumab combination therapy had similar efficacy in the PD-L1 positive subgroup compared to all participants. However, the combination therapy of atezolizumab with chemotherapy tends to increase the incidence of treatment-related AEs, especially immune-related AEs. Therefore, treatment-related AEs of atezolizumab should be monitored carefully and cautiously. Future primary studies are needed to identify key predictors associated with immune-related AEs as well as to compare their incidences to help the clinicians personalize treatment for patients with TNBC, working to improve their prognosis. Although atezolizumab has been withdrawn recently to be used in TNBC in the United States, the results of this MA may help the clinicians and manufacturers in the reassessment of atezolizumab and consideration of alternative ICIs in the treatment of TNBC.

## Supplementary Information

**Additional file 1: Supplementary Table S1.** Excluded studies with reasons. **Supplementary Figure S1.** Funnel plot showing risk of publication bias.

## Data Availability

Data are available in a public, open access repository. All data relevant to the study are included in the article or uploaded as supplementary information. The data used to support the findings of this study are included within the manuscript and supplementary files.
