# Supplementary material for: Combined atezolizumab and nab-paclitaxel in the treatment of triple negative breast cancer: a meta-analysis on their efficacy and safety
Source: BMC Cancer. 2022 Nov 5;22:1139. doi: 10.1186/s12885-022-10225-y (PMC9637314; doi:10.1186/s12885-022-10225-y)
Supplement: Supplementary file 1 — Additional file 1: Supplementary Table S1. Excluded studies with reasons. Supplementary Figure S1. Funnel plot showing risk of publication bias. [file 12885_2022_10225_MOESM1_ESM.docx]

Supplementary Table S1: Excluded studies with reasons

| **Study** | **Title** | **Reason for exclusion** |
| --- | --- | --- |
| NCT02322814 (8) | A study of cobimetinib plus paclitaxel, cobimetinib plus atezolizumab plus paclitaxel, or cobimetinib plus atezolizumab plus nab-paclitaxel as initial treatment for participants with triple negative breast cancer that has spread | Results are same with Brufsky, 2021 |
| Emens et al. 2019 (9) | Long-term clinical outcomes and biomarker analyses of atezolizumab therapy for patients with metastatic triple-negative breast cancer | No comparison with nab-paclitaxel group. |
| Adam et al. 2019 (10) | Atezolizumab plus nab-paclitaxel in the treatment of metastatic triple-negative breast cancer with 2-year survival follow-up | Results are same with Schmid, 2018 and Schmid, 2020 (IMpassion130) |
| Adam et al. 2020 (11) | Patient-reported outcomes from the phase III IMpassion130 trial of atezolizumab plus nab-paclitaxel in metastatic triple-negative breast cancer | Outcomes of trials are not reported and reported patient-reported outcomes only. |
| Emens et al. 2021 (12) | Atezolizumab and nab-paclitaxel in advanced triple-negative breast cancer: Biomarker evaluation of the IMpassion130 study | Reports were on based on low and high PD-L1 status and other biomarker status |
| Wang et al. 2020 (13) | Quantitative systems pharmacology model predictions for efficacy of atezolizumab and nab- paclitaxel in triple- negative breast cancer | It is virtual trial study rather than RCT |

| 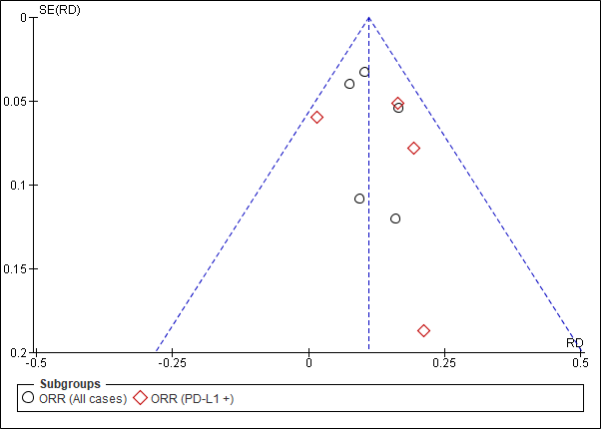 |
| --- |
| **Supplementary Figure S1:** Funnel plot showing risk of publication bias |
